# Sensitive Diagnosis and Post-Treatment Follow-Up of *Schistosoma mansoni* Infections in Asymptomatic Eritrean Refugees by Circulating Anodic Antigen Detection and Polymerase Chain Reaction

**DOI:** 10.4269/ajtmh.21-0803

**Published:** 2022-02-28

**Authors:** Pytsje T. Hoekstra, Afona Chernet, Claudia J. de Dood, Eric A. T. Brienen, Paul L. A. M. Corstjens, Niklaus D. Labhardt, Beatrice Nickel, Linda J. Wammes, Govert J. van Dam, Andreas Neumayr, Lisette van Lieshout

**Affiliations:** ^1^ Department of Parasitology, Leiden University Medical Center, Leiden, The Netherlands;; ^2^ Swiss Tropical and Public Health Institute, Basel, Switzerland;; ^3^ University of Basel, Basel, Switzerland;; ^4^ Department of Cell and Chemical Biology, Leiden University Medical Center, Leiden, The Netherlands;; ^5^ Department of Infectious Diseases and Hospital Epidemiology, University Hospital Basel, Basel, Switzerland;; ^6^ Department of Medical Microbiology, Leiden University Medical Center, Leiden, The Netherlands;; ^7^ Department of Public Health and Tropical Medicine, College of Public Health, Medical and Veterinary Sciences, James Cook University, Queensland, Australia

## Abstract

The increasing number of refugees coming from or passing through *Schistosoma*-endemic areas and arriving in Europe highlights the importance of screening for schistosomiasis on arrival, and focuses attention on the choice of diagnostic test. We evaluate the diagnostic performance of circulating anodic antigen (CAA) detection in 92 asymptomatic refugees from Eritrea. Results were compared with already-available stool microscopy, serology, and urine point-of-care circulating cathodic antigen (POC-CCA) data. For a full diagnostic comparison, real-time polymerase chain reaction (PCR) and the POC-CCA were included. All outcomes were compared against a composite reference standard. Urine and serum samples were subjected to the ultra-sensitive and highly specific up-converting particle lateral flow CAA test, *Schistosoma *spp. real-time PCR was performed on urine and stool, and the POC-CCA was used on urine using the G-score method. CAA was detected in 43% of urine and in 40% of serum samples. Urine PCR was negative in all 92 individuals, whereas 25% showed *Schistosoma* DNA in stool. POC-CCA was positive in 30% of individuals. The CAA test confirmed all microscopy positives, except for two cases that were also negative by all other diagnostic procedures. Post-treatment, a significant reduction in the number of positives and infection intensity was observed, in particular regarding CAA levels. Our findings confirm that microscopy, serology, and POC-CCA lack the sensitivity to detect all active *Schistosoma* infections. Accuracy of stool PCR was similar to microscopy, indicating that this method also lacks sensitivity. The CAA test appeared to be the most accurate method for screening active *Schistosoma* infections and for monitoring treatment efficacy.

## INTRODUCTION

Schistosomiasis affects more than 230 million individuals worldwide and is mainly endemic in sub-Saharan Africa. ^1^ The increasing number of African refugees and migrants traveling to Europe from or passing through *Schistosoma*-endemic regions stresses the importance of screening for *Schistosoma* infections, ^2^ but at the same time raises the question of which diagnostic test is most suitable for this specific population. Although the commercially available point-of-care (POC) test detecting circulating cathodic antigen (CCA) is being used more often for diagnosing *S. mansoni* infection in non-endemic study settings, ^3^^–^ ^7^ the detection of *Schistosoma*-specific antibodies remains the most widely used and recommended first-line test for screening, ^8^^,^ ^9^ whereas the detection of eggs in urine or stool is still often used as a reference standard to confirm infection as well as to determine the species. ^10^

These diagnostic methods, however, lack the sensitivity to detect infections of low intensity. In particular, in the case of refugees and migrants, diagnosing schistosomiasis is challenging because microscopy is often negative when performed on a single stool sample, and antibody detection is unable to differentiate between active and past infection. An alternative and more sensitive method for the diagnosis of imported *Schistosoma* infections in routine laboratory settings is the detection of *Schistosoma*-specific DNA. For example, *Schistosoma* internal transcriber-spacer-2 (ITS2) real-time polymerase chain reaction (PCR) has been shown to detect egg-negative cases and demonstrate that individual DNA loads decrease after treatment, ^11^ indicating its suitability to monitor efficacy of treatment. In addition, a significant number of microscopy-negative cases have detectable levels of circulating anodic antigen (CAA), a unique gut-associated antigen that is excreted continuously by adult worms and is therefore detectable within weeks of infection as well as in chronic infections. ^12^^–^ ^14^ CAA is detected by highly sensitive and specific monoclonal antibodies, ^15^ making the up-converting particle (UCP) lateral flow (LF) CAA test highly specific. The presence of CAA in urine or serum demonstrates the presence of living worms, ^13^ indicating active infection. CAA levels decline rapidly (within weeks) after treatment with praziquantel, ^16^^,^ ^17^ making this test suitable for both individual and community screening as well as for follow-up after praziquantel treatment. The UCP-LF CAA test is a genus-specific test able to identify active *Schistosoma* infections of all known species. ^13^ This test has shown high accuracy in detecting the four major *Schistosoma* species in different endemic ^13^^,^ ^18^^–^ ^21^ as well as non-endemic routine diagnostic settings. ^16^^,^ ^22^^–^ ^24^

We evaluated the clinical diagnostic value of the UCP-LF CAA test for diagnosing *Schistosoma* infections among asymptomatic East African refugees and migrants within 2 years after their arrival in Switzerland, and compared this to currently used routine diagnostics consisting of microscopic detection of eggs in stool and urine and antibody detection. ^25^^,^ ^26^ For a full diagnostic comparison, DNA detection by real-time PCR in stool and urine samples and the POC-CCA were included, and outcomes were compared against a composite reference standard. In addition, previous studies have shown that *Schistosoma* circulating antigens decrease after treatment, but at the same time suggest that the standard dose of praziquantel may not kill all worms present. ^12^ Therefore, this diagnostic comparison study includes not only the detection of infected cases, but also evaluates the UCP-LF CAA test for monitoring the efficacy of praziquantel treatment.

## MATERIALS AND METHODS

This was a nested study within the original study, which focused on assessing infectious and non-communicable health conditions among East African refugees at arrival and post-integration in Switzerland. ^7^^,^ ^25^^,^ ^26^ Ethical approval was obtained from the institutional research commission of the Swiss Tropical and Public Health Institute (Swiss TPH; ref. no. FK 120) and the ethics committee of Northwest and Central Switzerland (ref. no. EKNZ 2015-353). For the nested study, a complete baseline sample set—consisting of stool, urine, and serum samples—was available from 92 asymptomatic Eritrean refugees (87 men; median age, 26 years; age range, 18–63 years). At the Swiss TPH, stool samples were subjected to microscopy using a large-volume sedimentation technique to maximize the yield of detectable eggs. Serology consisted of three in-house assays that are used routinely at the Swiss TPH diagnostic center, the details of which have been described previously. ^25^ In addition, urine samples were tested using the POC-CCA according to manufacturer instructions; visible lines were documented according to their intensity as weak positive or clearly positive. Individuals who were found positive by any of these methods were treated with two doses of praziquantel (60 mg/kg body weight/dose) and monitored after approximately 12 to 18 months, when the same diagnostic procedures were repeated. ^26^ A complete post-treatment sample set—consisting of stool, urine, and serum samples—was available from 23 individuals (22 men; median age, 27 years; age range, 18–44 years).

At the Leiden University Medical Center (LUMC), samples were subjected to CAA detection (urine and serum), as well as DNA detection (urine and stool) and CCA detection (urine). Detection of CAA in urine and serum samples was performed using the laboratory-based, and most sensitive, concentration format of the UCP-LF CAA test, as described previously. ^13^^,^ ^18^ For urine samples the dry format of the test was used; serum samples were subjected to the wet format, which includes an additional UCP sonication step. ^13^ A reference standard of samples with a known CAA concentration was included to quantify individual CAA levels and to validate the cutoff (0.2 pg/mL for the urine test [UCAA*hT*3333] and 1 pg/mL for the serum test [SCAA500] as described in Corstjens et al. ^13^). Samples were considered positive if the CAA concentration exceeded this cutoff; samples below the cutoff were considered negative.

The *Schistosoma *genus-specific ITS2 real-time PCR was used to detect *Schistosoma* DNA in urine and stool samples, as described previously. ^27^^,^ ^28^ Since its implementation in 2019, we scored 100% for the *Schistosoma* PCR at the annual international helminths external quality assessment scheme provided by the Dutch Foundation for Quality Assessment in Medical Laboratories based on the distribution of genuine clinical samples. ^29^ The stool PCR output consisted of a cycle threshold value, which represented the amplification cycle in which the level of fluorescent signal exceeded the background fluorescence and thereby indicated the presence of parasite-specific DNA in the sample that was tested.

To determine the reproducibility of the POC-CCA test and to evaluate a more standardized scoring method, the test was repeated at LUMC using the same batch that also had been used at the Swiss TPH ^25^^,^ ^26^ (batch no. 50182; Rapid Medical Diagnostics, Pretoria, South Africa), which was still within the expiration date. The test was performed according to the manufacturer’s instructions, but now using the G-score scoring method for a more standardized interpretation and quantification of the test outcome. ^30^ POC-CCA G-scores were transformed into the more frequently used visual scores of trace (G2–3), 1+ (G4–5), 2+ (G6–7), and 3+ (G8–10). ^29^ POC-CCA traces were considered negative for the analysis. ^31^

Data were entered into a Microsoft Office 365 Excel spreadsheet (Microsoft Corp., Redmond, WA) and analyzed using GraphPad Prism 8.4.2 (GraphPad Software Inc., San Diego, CA) and SPSS version 25 (IBM Corp., Armonk, NY). Statistical analysis was performed using descriptive statistics. The agreement between the pre-treatment outcomes of the different diagnostic tests was determined by κ statistics. McNemar’s χ ^2^ test was used to compare sensitivity and specificity between diagnostic methods. In the absence of a single suitable reference standard, the sensitivity and specificity of the diagnostic tests were determined against a composite reference standard. To obtain the highest specificity, the diagnostic tests included in the composite reference standard were stool microscopy, urine CAA, serum CAA, and stool PCR. In this study, an individual was considered to be infected if either microscopy was positive or if at least two of the other diagnostic tests (i.e., urine/serum CAA, stool PCR) were positive. Spearman’s ρ was used to assess the relationship between CAA levels in urine and serum, as well as the relationship between CAA levels (urine/serum) and POC-CCA G-scores and PCR cycle threshold values.

## RESULTS

### Pre-treatment (*N* = 92).

**Figure 1.**
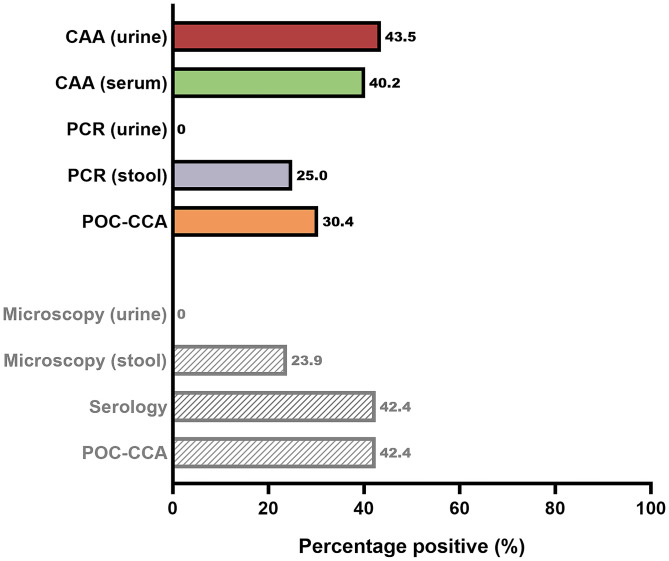
Percentage positive by urine and serum circulating anodic antigen (CAA), stool polymerase chain reaction (PCR), and point-of-care circulating anodic antigen (POC-CCA) (in color) compared with percentage positive by stool sedimentation microscopy, serology, and POC-CCA (Swiss Tropical and Public Health Institute, in gray) in a group of 92 asymptomatic Eritrean refugees. This figure appears in color at www.ajtmh.org.

Figure 1 presents an overview of the percentage of positive results of the different diagnostic tests performed. The greatest number of positives was observed with the UCP-LF CAA test; 43.5% of individuals had detectable CAA levels in urine and 40.2% of individuals had detectable CAA levels in serum. At the Swiss TPH, in 23.9% of individuals, *S. mansoni* eggs were observed in stool samples, whereas no eggs were found in urine samples and, based on antibody detection, 42.4% of individuals were positive. In addition, at the LUMC, DNA was detected in the stool samples of 25.0% of individuals, whereas no DNA was detected in urine samples and POC-CCA was positive in 30.4% of individuals.

**Figure 2.**
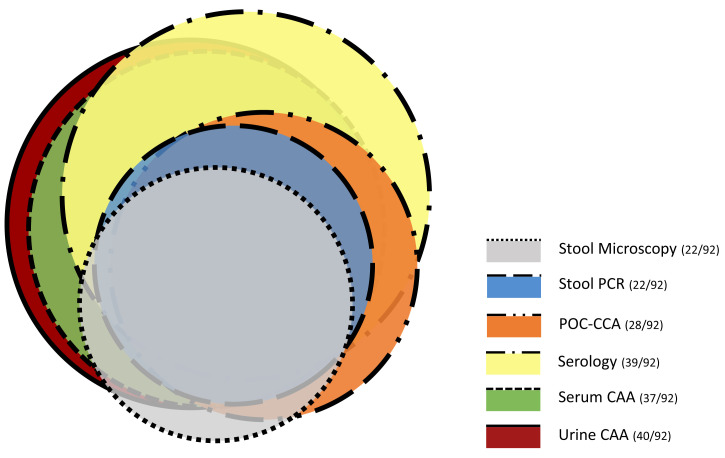
Proportional Venn diagram of urine and serum circulating anodic antigen (CAA) positives compared with stool sedimentation microscopy, serology, stool polymerase chain reaction (PCR), and point-of-care circulating anodic antigen (POC-CCA) positives in a group of 92 asymptomatic Eritrean refugees. This figure appears in color at www.ajtmh.org.

The agreement between urine and serum CAA and stool microscopy, serology, and stool PCR and POC-CCA is shown in Figure 2. In total, 48 individuals (52.2%) were positive by at least one of these five diagnostic tests. The majority of stool microscopy positives was detected by urine and/or serum UCP-LF CAA, stool PCR, or POC-CCA. Almost half of the CAA positive cases were not detected by stool microscopy nor by stool PCR, whereas the POC-CCA positives showed more overlap with the CAA positives. The degree of agreement as well as the discordance between the UCP-LF CAA test, stool PCR, and POC-CCA are shown in Table 1. Additional analyses of the degree of agreement and the discordance compared with the Swiss TPH data of stool microscopy and serology are included in Supplemental Table 1. The correlation between the UCP-LF CAA test, stool PCR, and POC-CCA is shown in Supplemental Figure S1 and Supplemental Table S2.

**Table 1:** The level of agreement between circulating anodic antigen (CAA), stool polymerase chain reaction (PCR), and point-of-care circulating cathodic antigen (POC-CCA) by Cohen’s k coefficient and McNemar’s xAU26 2 test in a group of 92 asymptomatic Eritrean refugees

Diagnostic test	Reference test *n*		Cohen’s κ			McNemar’s *P *value
			κ value	Interpretation*	*P* value	
	CAA (urine)					
CAA (serum)	Positive	Negative				
Positive	36	1	0.89	Almost perfect	< 0.001	0.375
Negative	4	51				
	PCR (stool)					
CAA (urine)	Positive	Negative				
Positive	22	18	0.56	Moderate	< 0.001	< 0.001
Negative	1	51				
	PCR (stool)					
CAA (serum)	Positive	Negative				
Positive	22	15	0.61	Substantial	< 0.001	0.001
Negative	1	54				
	POC-CCA					
CAA (urine)	Positive	Negative				
Positive	23	17	0.50	Moderate	< 0.001	0.017
Negative	5	47				
	POC-CCA					
CAA (serum)	Positive	Negative				
Positive	24	13	0.60	Moderate	< 0.001	0.049
Negative	4	51				
	PCR (stool)					
POC-CCA	Positive	Negative				
Positive	18	10	0.60	Moderate	< 0.001	0.302
Negative	5	59				

CAA = circulating anodic antigen; PCR = polymerase chain reaction; POC-CCA = point-of-care circulating cathodic antigen.

* Interpretation of κ coefficient: ≤0, chance; 0.01 to 0.20, slight; 0.21 to 0.40, fair; 0.41 to 0.60, moderate; 0.61 to 0.80, substantial; 0.81 to 0.99, almost perfect.

The sensitivity and specificity of all diagnostic tests compared with the composite reference standard are shown in Table 2. In total, 39 individuals (42.4%) were positive by the composite reference standard. The UCP-LF CAA test in serum showed the highest sensitivity (95%), followed by the UCP-LF CAA test in urine (92%). The POC-CCA test showed a lower sensitivity (62%), comparable to stool PCR (56%) and stool microscopy (56%).

**Table 2:** Accuracy of the different diagnostic tests compared against a composite reference standard in a group of 92 asymptomatic Eritrean refugees

Diagnostic test and location	Outcome	CRS*, *n*		Diagnostic accuracy, %		Cohen’s κ			McNemar’s *P* value
		Positive	Negative	Sensitivity	Specificity	κ value	Interpretation†	*P* value	
LUMC, Leiden, the Netherlands									
CAA (urine)	Positive	36	4	92	92	0.845	Almost perfect	< 0.001	1.000
	Negative	3	49						
CAA (serum)	Positive	37	0	95	100	0.955	Almost perfect	< 0.001	0.500
	Negative	2	53						
PCR (stool)‡	Positive	22	1	56	98	0.576	Moderate	< 0.001	< 0.001
	Negative	17	52						
POC-CCA§	Positive	24	4	62	92	0.561	Moderate	< 0.001	0.019
	Negative	15	49						
Swiss TPH, Basel, Switzerland‖									
Microscopy (stool)¶	Positive	22	0	56	100	0.599	Moderate	< 0.001	< 0.001
	Negative	17	53						
Serology	Positive	31	8	79	85	0.644	Substantial	< 0.001	1.000
	Negative	8	45						
POC-CCA§	Positive	29	10	74	81	0.555	Moderate	< 0.001	1.000
	Negative	10	43						

CAA = circulating anodic antigen; CRS = composite reference standard; LUMC = Leiden University Medical Center; PCR = polymerase chain reaction; POC-CCA = point-of-care circulating cathodic antigen; Swiss TPH = Swiss Tropical and Public Health Institute.

* The CRS was based on the detection of eggs in stool and/or CAA in urine/serum and/or DNA in stool. An individual was considered positive if either microscopy was positive or at least two of the other diagnostic tests were positive.

† Interpretation of κ coefficient: ≤0, chance; 0.01 to 0.20, slight; 0.21 to 0.40, fair; 0.41 to 0.60, moderate; 0.61 to 0.80, substantial; 0.81 to 0.99, almost perfect.

‡ All urine samples were negative by urine PCR.

§ The same POC-CCA batch was used at LUMC and Swiss TPH (no. 50182), but a different scoring approach was used; see Materials and Methods.

‖ Data available from Swiss TPH.

¶ All urine samples were negative by urine microscopy.

### Post-treatment (*N* = 23).

The total number of positive participants decreased after treatment, as detected by urine CAA (21.7%), serum CAA (17.4%), stool PCR (8.7%), and POC-CCA (8.7%). A significant decrease in average CAA levels in urine and serum was observed after treatment, with intensity reduction rates of 95.0% and 93.6% respectively. Individual pre- and post-treatment outcomes of urine and serum CAA, stool PCR, and POC-CCA are shown in Figure 3. The majority of individuals became negative after treatment. One individual remained positive by all four diagnostic methods (indicated by the green shapes in Figure 3), whereas another individual remained positive by serum CAA and stool PCR only (indicated by the red shapes in Figure 3B and 3C).

**Figure:**
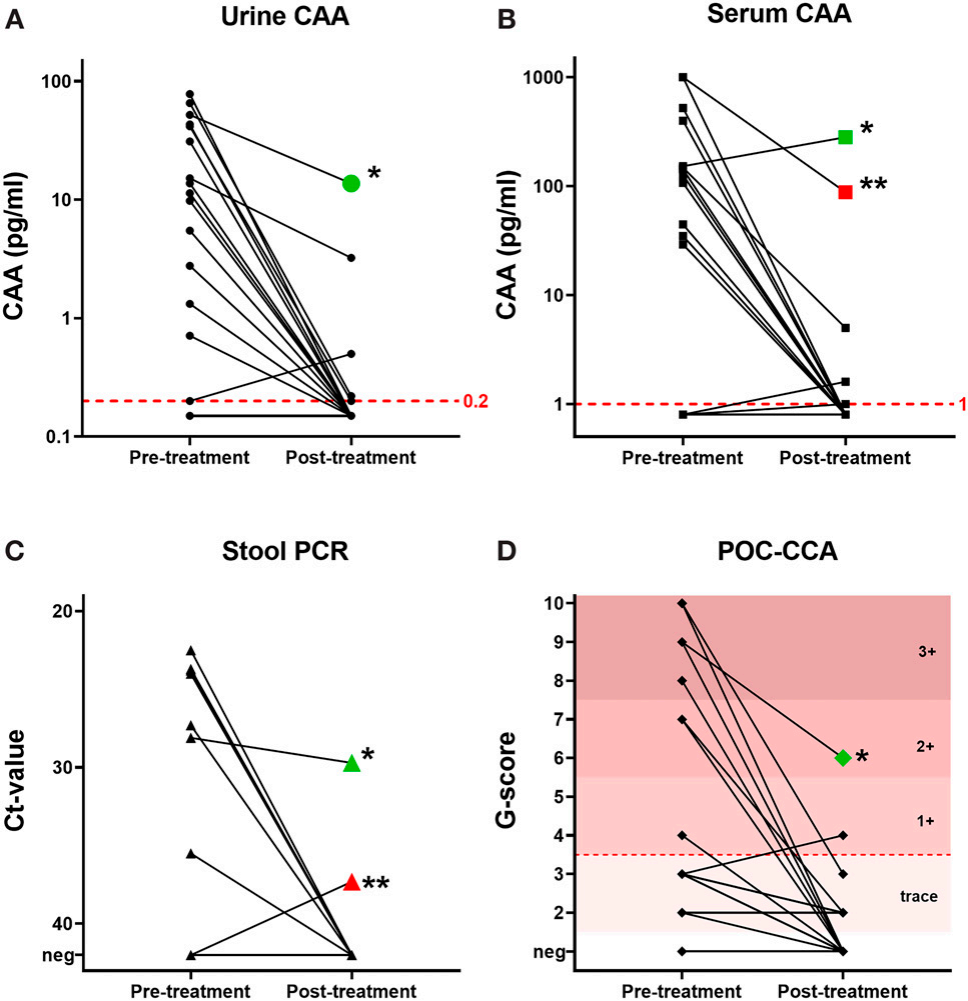
Figure 3. Effect of treatment on individual test outcomes of (**A**) urine circulating anodic antigen (CAA), (**B**) serum CAA, (**C**) stool polymerase chain reaction (PCR), and (**D**) point-of-care circulating anodic antigen (POC-CCA) in a group of 23 asymptomatic Eritrean refugees. Ct-value = cycle threshold value. * Individual who tested positive in all four tests after treatment (green shapes). ** Individual who tested positive by serum CAA and stool PCR after treatment (red shapes). This figure appears in color at www.ajtmh.org.

## DISCUSSION

The high prevalence of *S. mansoni* infections in this group of asymptomatic Eritrean refugees, as demonstrated previously by stool microscopy, serology, and POC-CCA, was confirmed in our study by the highly sensitive and specific UCP-LF CAA test, stressing the importance of timely and efficacious screening of refugees and migrants for *Schistosoma* infections.

Previous results from the main study suggested that a combination of POC-CCA and serology would be the ideal screening method for *S. mansoni* infections in asymptomatic refugees. ^25^ In our study, the UCP-LF CAA test was evaluated as a potential new tool for screening and post-treatment monitoring of *Schistosoma* infections. Our results demonstrate that a single urine or serum UCP-LF CAA test detected an even greater number of *Schistosoma* positives compared with stool microscopy and serology. The UCP-LF CAA test was able to confirm all microscopy positives, except two cases that were positive only by microscopy and negative by all other diagnostic tests. In addition, 21 positives were observed by the UCP-LF CAA test that were negative by microscopy, confirming the added value of CAA detection in this specific population. Especially in the case of low worm burden the detection of eggs can be very difficult, ^32^^,^ ^33^ while living worms continue to release CAA which is detected by the UCP-LF CAA test. In our study, observed CAA levels were relatively high, in particular given the fact that none of the individuals showed any schistosomiasis-related signs or symptoms. ^25^ Because CAA is excreted by all *Schistosoma* species, even in the case of hybrid infections, ^24^ the UCP-LF CAA test is applicable to migrants originating from all *Schistosoma*-endemic areas. Extensive parasitological diagnosis has been performed in this cohort and there was no evidence of other helminth infections, ^7^ which is in line with the high specificity of the UCP-LF CAA test.

In the absence of a single suitable reference standard, the diagnostic methods were compared against a composite reference standard, ^34^^,^ ^35^ which is an approach that has also been used previously. ^17^^,^ ^19^ It can serve as an alternative to, for example, latent class analysis, as criteria for this type of analysis are often difficult to meet, especially in the case of schistosomiasis diagnostic evaluation studies (e.g., conditional dependence among tests, small sample size, the limited number of tests included in the analysis). ^36^ The urine UCP-LF CAA test showed a sensitivity of 92% compared with the composite reference standard, which is similar to findings from previous studies, even though they were performed in endemic settings and some of them concerned other *Schistosoma* species. ^19^^,^ ^20^^,^ ^37^ Compared with the composite reference standard, the UCP-LF CAA test in serum demonstrated the highest sensitivity of 95%, thereby signifying its potential use for screening purposes for accurate detection and monitoring of *Schistosoma* infections in refugees and migrants. Although the performance of stool PCR was expected to be better than microscopy, as also observed in previous studies, our results show a similar sensitivity. An explanation for this could be that a large-volume sedimentation technique with at least 10 g of stool was used to detect the eggs, resulting in a substantially higher sensitivity than a single Kato-Katz thick smear, as often used in endemic settings. ^38^ Although the absolute number of positive cases was similar by either CAA or serology, the diagnostic accuracy of serology was substantially less compared with that of the UCP-LF CAA test. This seems to be a combination of 1) serology missing some cases with an active infection based on the presence of CAA and 2) persisting positive serology when no active infection could be demonstrated. This inability of serology to differentiate between active and passive infection is well known. ^39^

CAA levels in urine and serum decreased significantly after treatment, indicating clearance of infection and thereby again confirming the specificity of the UCP-LF CAA test. Although the praziquantel treatment regimen (two doses of 60 mg/kg body weight) was aimed at parasite eradication rather than parasite reduction (standard single dose of 40 mg/kg recommended for mass drug administration in endemic regions by the WHO), in some individuals, urine and serum CAA levels remained detectable 12 to 18 months after treatment. Reinfection can be ruled out as individuals remained in Switzerland during the study period. ^26^ Because CAA levels decreased in most cases after treatment, and because treatment compliance was not controlled (no directly observed treatment), these individuals might not have complied fully with the treatment schedule of two doses of praziquantel, thereby resulting in an uncleared infection. Because CAA levels decrease (within weeks) after treatment, ^12^^,^ ^16^^,^ ^17^ short-term follow-up is possible. This would be a major advantage especially for refugees or migrants, because, in general, they are a very mobile population often resulting in lower compliance and greater lost-to-follow-up rates.

When looking at the reproducibility and performance of the POC-CCA test, a comparable percentage of positives was observed at both the Swiss TPH and LUMC, demonstrating the consistent performance of this specific POC-CCA batch and confirming that testing after shipping and storing the urine samples frozen for 1 year does not influence the outcome of the test when using the same batch. Unfortunately, although the same batch was used, it was difficult to compare the individual results because of the difference in the scoring method; the POC-CCA test at the Swiss TPH was only scored (weak) positive or negative. ^25^ A moderate κ agreement and a strong correlation was found between the POC-CCA test performed at the LUMC and the other diagnostic tests (stool microscopy, stool PCR, serum/urine CAA), whereas only a slight to moderate κ agreement between these diagnostic tests and the POC-CCA test performed at the Swiss TPH was found. A clear effect of treatment was observed based on G-scores, whereas at the Swiss TPH, several inconclusive and inconsistent results were observed at follow-up. ^26^ Overall, this comparison stresses the necessity of using a standardized method when scoring POC-CCA test outcomes pre-treatment as well as post-treatment.

Because the UCP-LF CAA test is a genus-specific test, ^13^ the results of our study cannot confirm whether the CAA test only detected *S. mansoni* or also other *Schistosoma* infections. However, based on microscopy, only *S. mansoni* eggs were detected in this study population, which is confirmed by the presence of *Schistosoma* DNA in stool samples and the absence of any *Schistosoma* DNA in urine samples. Furthermore, considering their area of origin (Eritrea) and migration route, only *S. mansoni* infections were assumed in this group. ^25^

It was not possible to determine the correlation between intensity of infection based on stool microscopy and diagnostic tests performed at the LUMC because of the absence of fecal egg counts, but such a correlation has been described in previous studies. ^25^^,^ ^40^^–^ [Bibr b41] ^42^

Unfortunately, a complete post-treatment follow-up data set was only available from a relatively small number of individuals. In addition, only those who tested positive at baseline (pre-treatment) were monitored after treatment. For better comparison, follow-up could have taken place in all those who participated in the initial study and, preferably, also earlier than 12 to 18 months after treatment.

The UCP-LF CAA test is not commercially available yet. At the moment, the test is used for research purposes and in collaborative projects. However, recently the UCP-LF CAA test has been implemented into the routine diagnostic laboratory of the LUMC and is now available for individual case detection. In addition, initiatives have been taken to develop a more easy-to-use and visually scored POC-CAA test based on a finger-prick blood sample (https://www.finddx.org/ntd/schisto-rdts/).

## CONCLUSION

This study confirms the importance of screening (asymptomatic) refugees and migrants for schistosomiasis. The accuracy of stool PCR was similar to extensive microscopy, indicating that this method also lacks sensitivity in this specific population. The UCP-LF CAA assay appears to be the most sensitive method for screening *Schistosoma* infections. In addition, although tested in only a small number of individuals, our results also confirm CAA to be a suitable, genus-specific marker for monitoring praziquantel treatment efficacy.

## Supplemental Material


Supplemental materials
