# Supplementary material for: Sensitive Diagnosis and Post-Treatment Follow-Up of Schistosoma mansoni Infections in Asymptomatic Eritrean Refugees by Circulating Anodic Antigen Detection and Polymerase Chain Reaction
Source: Am J Trop Med Hyg. 2022 Feb 28;106(4):1240–6. doi: 10.4269/ajtmh.21-0803 (PMC8991328; doi:10.4269/ajtmh.21-0803)
Supplement: Supplementary file 1 [file tpmd210803.SD1.pdf]

Supplemental Table 1. The level of agreement between CAA, stool PCR and POC-CCA and routine diagnostic tests performed at Swiss TPH (stool sedimentation microscopy and serology) by Cohen's kappa coefficient and McNemar's χ^2-test in a group of 92 asymptomatic Eritrean refugees.

N=92			Cohen's kappa κ	Interpretation[1]	*p*-value	McNemar *p*-value
	**Microscopy (stool)**					
**CAA (urine)**	Positive	Negative				
Positive	19	21	0.440	Moderate	$p<0.001$	$p<0.001$
Negative	3	49				
	**Microscopy (stool)**					
**CAA (serum)**	Positive	Negative				
Positive	20	17	0.540	Moderate	$p<0.001$	$p=0.001$
Negative	2	53				
	**Microscopy (stool)**					
**PCR (stool)**	Positive	Negative				
Positive	18	5	0.735	Substantial	$p<0.001$	$p=1.000$
Negative	4	65				
	**Microscopy (stool)**					
**POC-CCA**	Positive	Negative				
Positive	18	10	0.618	Substantial	$p<0.001$	$p=0.180$
Negative	4	60				
	**Serology**					
**CAA (urine)**	Positive	Negative				
Positive	30	10	0.579	Moderate	$p<0.001$	$p=1.000$
Negative	9	43				
	**Serology**					
**CAA (serum)**	Positive	Negative				
Positive	30	7	0.642	Substantial	$p<0.001$	$p=0.804$
Negative	9	46				
	**Serology**					
**PCR (stool)**	Positive	Negative				
Positive	21	2	0.529	Moderate	$p<0.001$	$p<0.001$
Negative	18	51				
	**Serology**					
**POC-CCA**	Positive	Negative				
Positive	23	5	0.515	Moderate	$p<0.001$	$p=0.027$
Negative	16	48				

1. Interpretation of κ coefficient: ≤0, chance; 0.01-0.20, slight; 0.21-0.40, fair; 0.41-0.60, moderate; 0.61-0.80, substantial; 0.81-0.99, almost perfect.

Abbreviations: CAA, circulating anodic antigen; PCR, polymerase chain reaction; POC-CCA, point-of-care circulating anodic antigen.

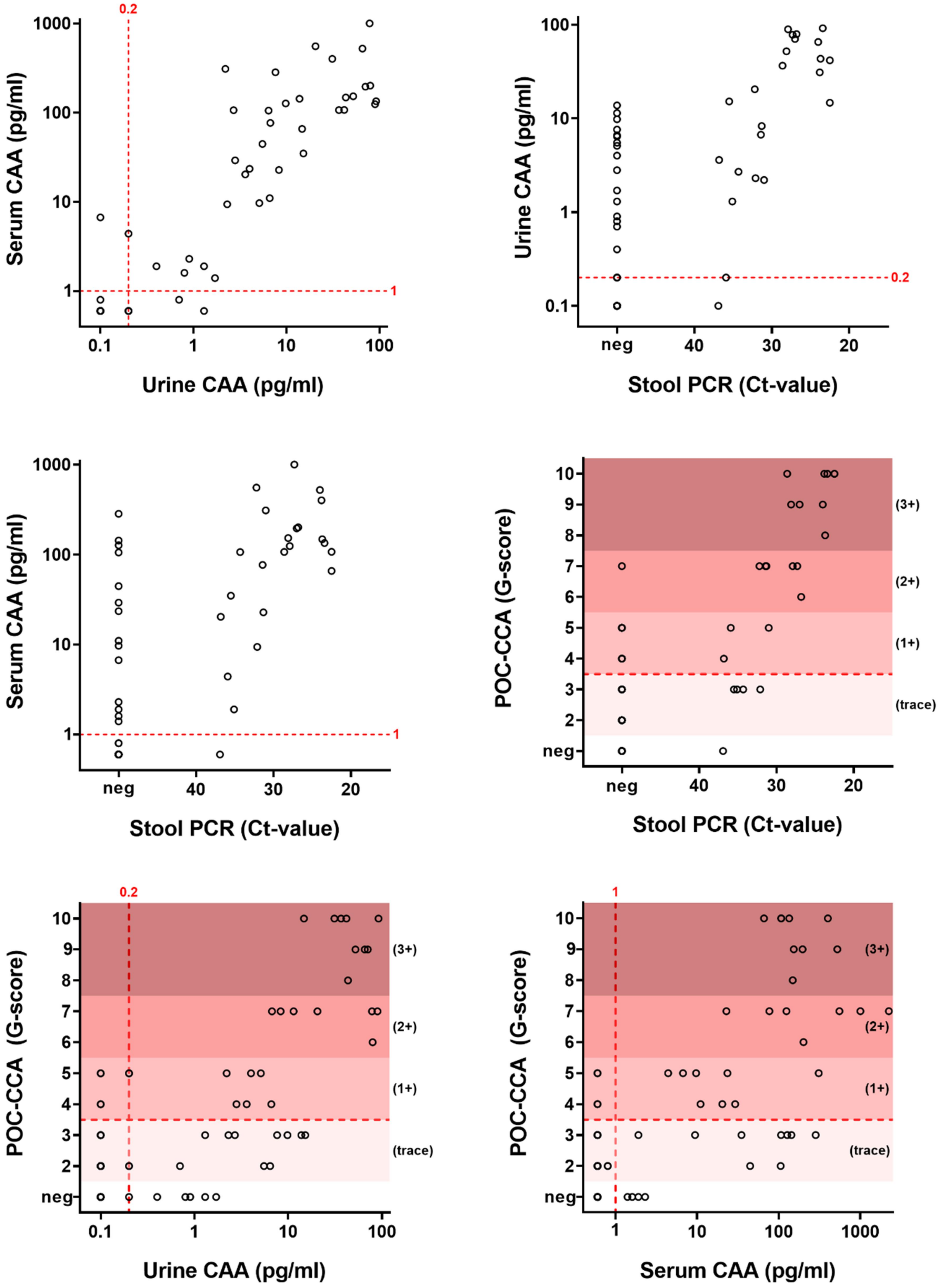

Supplemental Figure 1. Pre-treatment correlation between CAA, stool PCR and POC-CCA in a group of 92 asymptomatic Eritrean refugees.

Abbreviations: CAA, circulating anodic antigen; Ct-value, cycle threshold value; PCR, polymerase chain reaction; POC-CCA, point-of-care circulating anodic antigen.

Supplemental Table 2. Pre-treatment correlation between CAA, stool PCR and POC-CCA in a group of 92 asymptomatic Eritrean refugees.

Diagnostic comparison	Spearman's rho[1]	*p*-value	Interpretation
Serum CAA vs Urine CAA	0.874	p<0.01	Very strong
Urine CAA vs Stool PCR	-0.659	p<0.01	Strong
Serum CAA vs Stool PCR	-0.636	p<0.01	Strong
Urine CAA vs POC-CCA	0.611	p<0.01	Strong
Serum CAA vs POC-CCA	0.683	p<0.01	Strong
POC-CCA vs Stool PCR	-0.676	p<0.01	Strong

1. Interpretation of Spearman's rho: ≤0, chance; 0.00 -0.20, negligible; 0.21-0.40, weak; 0.41-0.60, moderate; 0.61-0.80, strong; 0.81-1.00, very strong.

Abbreviations: CAA, circulating anodic antigen; PCR, polymerase chain reaction; POC-CCA, point-of-care circulating anodic antigen.
